# Surgical treatment to multiple primary lung cancer patients: a systematic review and meta-analysis

**DOI:** 10.1186/s12893-019-0643-0

**Published:** 2019-12-03

**Authors:** Ting-Fei Chen, Chun-Ying Xie, Bing-Yu Rao, Shi-Chao Shan, Xin Zhang, Bo Zeng, Yi-Yan Lei, Hong-He Luo

**Affiliations:** Department of Thoracic Surgery, The First Affiliated Hospital, Sun Yat-sen University, Guangzhou, People’s Republic of China

**Keywords:** Multiple primary lung cancer, Surgery, Prognosis, Meta-analysis

## Abstract

**Background:**

As there is no consensus on the optimal surgery strategy for multiple primary lung cancer (MPLC), we conducted this study to address this issue by comparing the prognosis of MPLC patients underwent different surgical strategies including sublobar resection and the standard resection through a systemic review and meta-analysis.

**Methods:**

Relevant literature was obtained from three databases including PubMed, Embase and Web of Science. Inclusion and exclusion criteria were set for the screening of articles to be selected for further conduction of systemic review and meta-analysis. The HRs of OS of the sublobar group compared with standard resection group were extracted directly or calculated indirectly from included researches.

**Results:**

Ten researches published from 2000 to 2017 were included in this study, with 468 and 445 MPLC cases for the standard resection group and sublobar resection group respectively. The result suggested that OS of MPLC patients underwent sublobar resection (segmentectomy or wedge resection for at least one lesion) was comparable with those underwent standard resection approach (lobectomy or pneumonectomy for all lesions), with HR 1.07, 95% CI 0.67–1.71, *p* = 0.784. Further analysis found no difference in subgroups of synchronous and metachronous (from second metachronous lesion), different population region and dominant sex type.

**Conclusions:**

This study may reveal that sublobar resection is acceptable for patients with MPLC at an early stage, because of the equivalent prognosis to the standard resection and better pulmonary function preservation. Further research is needed to validate these findings.

## Background

It was reported that, as a special type of lung cancer, multiple primary lung cancer (MPLC) occurred on 0.2–20% of all lung cancer cases [1]. In 1975, Martini and Melamed [2] proposed the first diagnostic criteria for MPLC, which was widely accepted and used in the clinical field. In 2003, American College of Chest Physicians (ACCP) published the criteria for the diagnosis of MPLC, which was updated in 2007 and 2013. The new criteria took into account molecular genetic characteristics of cancers and elongated the interval to 4 years for the diagnosis of metachronous MPLC [3].

At present, surgery is considered as the first choice of treatments for MPLC patients [4, 5] as few researches concerning other treatments were reported. Although there were some studies about surgical treatment of MPLC, it remains controversial about the prognosis of these patients and its impacting factors, such as different resection methods [6]. No consensus has been reached on whether sublobar resection (segmentectomy or wedge resection) would bring along a worse prognosis compared with standard resection (anatomical lobectomy or pneumonectomy for all lesions) for MPLC patients. Hence, we looked into the prognosis of post-operative MPLC patients for further information and guidance to make clinical decisions better.

Guidelines such as NCCN, BTS, CSCO, etc. are used for clinical practice currently for lung cancer. According to the eighth edition of AJCC TNM staging system [7], tumor nodules located in the same lobe, ipsilateral lobes and contralateral lobes of the lung are defined as T3 M0, T4 M0 and M1a respectively. These nodules tend to be considered intrapulmonary metastasis rather than primary sites, which might include some MPLC that should actually be removed surgically. Some MPLC cases are neglected or assigned into T3/T4/M1a category. Aiming at this special kind of lung cancer, ACCP published an article concerning the diagnosis and treatment of MPLC [3]. However, no specific treatment strategy had been given in these guidelines or literature, especially for surgical resection methods. Therefore, the effect of different surgical methods on MPLC patients remains uncertain.

A few researches on ground glass opacity (GGO) suggested that those underwent lung-sparing resection could get better prognosis [8]. And most MPLC patients tend to be at an early stage, they share similarities with GGO. As there are more than one lesions, the anatomical resection on all lesions may not be practical on some MPLC patients because of pulmonary function loss. As we know, sublobar resection may preserve more pulmonary function than lobectomy and pneumonectomy. However, whether the sublobar resection method is advisable for MPLC patients remains controversial. So, we conducted a meta-analysis to evaluate prognostic factors of different surgical strategies on MPLC patients and to find out whether the sublobar resection is suitable for MPLC patients.

## Methods

### Search strategy

The comprehensive online searches were performed by two independent authors from three databases, including PubMed, Embase and Web of Science from the date of inception to Dec 2018. To achieve maximum sensitivity, we combined the terms “multiple primary lung cancer” or “second primary lung cancer” or “multifocal lung cancer” or “synchronous” or “metachronous” and “Lung Neoplasms/secondary or Lung Neoplasms/therapy” as either keywords or MeSH (Medical Subject Headings) terms. 3987 records were obtained from the three databases and 2 more from peers familiar with this field. After duplicate removal and rough screening, 48 articles were left for further assessment of full-text and 10 of them were selected for the qualitative and quantitative synthesis of this study. The screening process of this research followed the PRISMA statement [9].

### Selection and quality assessment

The inclusion criteria included: a) synchronous and/or metachronous MPLC cases were studied; b) the definition of MPLC criteria should be clearly stated c) two or more lesions were surgically removed in each case d) the study included OS (5-year at least) as the main outcome e) HR and 95%CI of OS according to sublobar and standard resection methods, or related tables or graphs for data extraction were provided.

The exclusion criteria were as follows. a) articles including abstracts, letters, case reports, reviews and non-clinical studies; b) articles written in languages other than English; c) studies including cases with primary malignant tumor of other organs; d) those had less than 10 cases within each research group; e) researches with low quality according Newcastle-Ottawa Scaling system.

Newcastle-Ottawa Scaling system was adopted for the quality assessment of literature, which was composed of three parts: selection (0–4 stars), comparability (0–2 stars) and outcome (0–3 stars). Articles achieving 6 or more stars were considered of high quality. The quality evaluation of each selected article was carried out by two independent researchers.

### Statistical analysis

Ln (HR) and SE (standard error) were used for data combination [10]. The HR and 95%CI were extracted from each article directly when provided, or calculated indirectly by data reading from Kaplan-Meier survival curve with Engauge Digitizer software.

Cochran’s Q test and Higgins *I*-squared test were used to evaluate the heterogeneity of included studies. And *p* < 0.10 or *I*^2^ > 50% was considered relatively high heterogeneity, in which case random-effect model would be applied. Subgroup analysis and sensitivity analysis were performed to find the source of heterogeneity. Publication bias was shown by funnel plot and examined quantitatively be Begg’s test and Egger’s test. Statistical analysis was carried out with R version 3.5.2 (R Foundation for Statistical Computing, Vienna, Austria) and its meta 4.9–4 package. *p* < 0.05 was considered statistically significant.

## Results

### Literature search

**Fig. 1:**
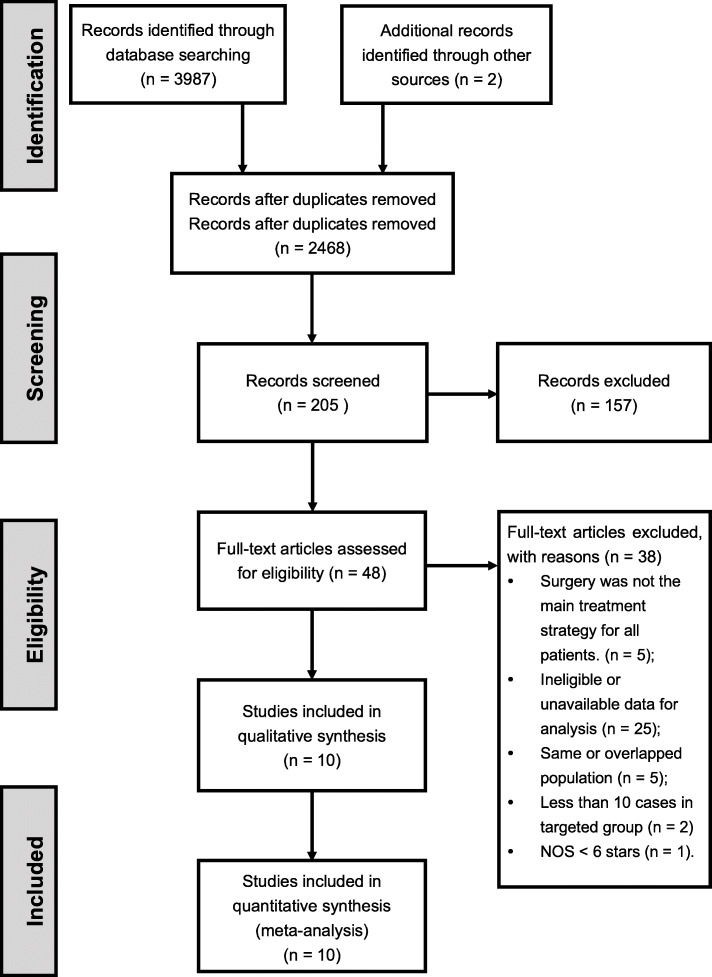
Flowchart based on PRISMA

In total 3987 relative articles were obtained from the three databases and after screening, ten studies published from 2000 to 2017 were included for further analysis (Fig. 1). The characteristics of these articles were shown in Table 1 as follows.

**Table 1:** Clinicopathologic characteristics of included studies

Study (Year)	Period	Country	Criteria	Type	Cases	Male (%)	Female (%)	Mean Age (years)	Smoker	Interval (months)	NOS Scale
Van Rens (2000) [11]	1970–1997	Netherlands	M-M	SYN	85	76 (89.4%)	9 (10.6%)	65.8	NA	NA	6
Doddoli (2001) [12]	1985–1999	France	M-M	MET	38	35 (92%)	3 (8%)	63 ± 8	37	60 ± 51	6
Trousse (2007) [13]	1985–2006	France	defined	SYN	125	98 (78.4%)	27 (21.6%)	61.5 ± 9.9	111	NA	7
De Leyn (2008) [14]	1990–2007	Belgium	defined	SYN	36	29 (80.6%)	7 (19.4%)	64.5	NA	NA	7
Riquet (2008) [15]	1993–2005	France	M-M	Mixed	234 SYN – 118 MET – 116	194 (82.9%)	40 (17.1%)	63.7 ± 9.1	NA	NA	6
Lee (2009) [16]	1995–2008	USA	defined	MET	58	23 (39.7%)	35 (60.3%)	67	NA	42	6
Voltolini (2010) [17]	1990–2007	Italy	defined	SYN	43	40 (93.0%)	3 (7.0%)	66.7 ± 6.9	40	NA	7
Hamaji (2013) [18]	2000–2009	USA	defined	MET	161	88 (54.7%)	73 (45.3%)	70	138	42.7	7
Ishikawa (2014) [19]	1995–2009	Japan	Modified M-M	SYN	93	36 (38.7%)	57 (61.3%)	68	33	NA	6
Xiao (2017) [20]	2004–2015	China	M-M	SYN	52	36 (69.2%)	16 (30.8%)	60.3 ± 8.61	38	NA	7

Notes: *M-M* Martini-Melamed criteria, *SYN* Synchronous, *MET* Metachronous, *NOS* Newcastle-Ottawa Scaling system

All ten articles compared the prognosis of MPLC patients treated surgically by standard and sublobar methods (Tables 1, 2, and 3). Those applying anatomical lobectomy or pneumonectomy for all sites were referred to as standard group, while cases adopting segmentectomy or wedge resection for at least one of the cancerous sites were named sublobar group. The total number of MPLC cases were 468 and 445 for standard and sublobar group respectively. Besides, in the research by Van Rens et al., the clinicopathological information given were based on 85 patients in total, while the survival analysis was carried out in 73 of them available for follow-up, whose characteristics were not different from the total group [11]. Most cases were at an early stage in terms of TNM staging system as reported. According to literature, 5.8–44% of MPLC patients experienced post-operative complications [12–15, 17, 18, 20] and the peri-operative death rates ranged from 0 to 14.1% [11–13, 15, 17–20]. The 5-year OS rates ranged from 19 to 87% [11–20]. Eight articles described the median survival time for post-operative MPLC cases, ranging from 30 to 72.9 months [11–15, 17, 18, 20].

**Table 2:** Clinicopathologic characteristics of included studies (continued)

Study (Year)	Location		Staging Criteria	TNM Stage		pN		Histology			
	Ipsilateral	Contralateral		I	II	pN0	pN1 + pN2	Identical	Different	ADK	SCC
Van Rens (2000) [11]	42 (49.4%)	43 (50.6%)	1997	40	29	NA	NA	50 (68.5%)	23 (31.5%)	30 (35.4%)	49 (57.6%)
Doddoli (2001) [12]	17 (44.7%)	21 (55.3%)	1997	27	4	NA	NA	23 (61.0%)	15 (39.0%)	28 (30.0%)	37 (49.0%)
Trousse (2007) [13]	91 (72.8%)	34 (27.2%)	1997	NA	NA	44	54	104 (83.2%)	21 (16.8%)	64 (51.2%)	36 (28.8%)
De Leyn (2008) [14]	0 (0.0%)	36 (100.0%)	1997	34		24	11 + 1	18 (50.0%)	18 (50.0%)	23 (31.9%)	38 (52.8%)
Riquet (2008) [15]	156 (66.7%)	78 (33.3%)	1997	75	20	144	31 + 59	135 (57.9%)	98 (42.1%)	109 (46.6%)	80 (34.2%)
Lee (2009) [16]	17 (29.0%)	41 (71.0%)	NA	56	1	NA	NA	42 (72.0%)	12 (21.0%)	95 (82.0%)	11 (9.0%)
Voltolini (2010) [17]	15 (35.0%)	28 (65.0%)	1997	8	28	25	9 + 9	27 (63.0%)	16 (37.0%)	28 (65.0%)	15 (25.0%)
Hamaji (2013) [18]	48 (29.8%)	113 (70.2%)	2002 & 2007	124	16	NA	NA	123 (76.4%)	38 (23.6%)	203 (63.0%)	75 (23.3%)
Ishikawa (2014) [19]	67 (72.0%)	26 (28.0%)	7th	75	9	75	10 + 8	93 (100.0%)	0 (0.0%)	93 (100.0%)	0 (0.0%)
Xiao (2017) [20]	28 (53.9%)	24 (46.2%)	7th	27	24	36	16 + 0	20 (38.5%)	32 (61.5%)	70 (66.0%)	34 (32.1%)

Notes: *NA* Not available, *ADK* Adenocarcinoma, *SCC* Squamous cell carcinoma

**Table 3:** Clinicopathologic characteristics of included studies (continued)

Study (Year)	Perioperative Morbidity	Perioperative Mortality	Resection		Adjuvant Therapy	Median Survival (months)			5-year OS		
			Sublobar	Standard		Total	Sublobar	Standard	Total	Sublobar	Standard
Van Rens (2000) [11]	NA	12 (14.1%)	32	41	NA	55.2	NA	NA	19%	NA	NA
Doddoli (2001) [12]	4 (11%)	5 (13%)	12	26	11 (29%)	31	NA	NA	32%	NA	NA
Trousse (2007) [13]	34 (27.2%)	6 (29.0%)	43	82	62 (49.6%)	34.932	NA	NA	34%	NA	NA
De Leyn (2008) [14]	16 (44.4%)	1 (2.8%)	26	10	6 (16.7%)	49.4	NA	NA	38.1%	NA	NA
Riquet (2008) [15]	66 (28.2%)	20 (8.6%)	54	180	62 (26.5%)	30	NA	NA	32.7%	36.4%	34.1%
Lee (2009) [16]	NA	NA	35	23	NA	NA	NA	NA	66%	59%	75%
Voltolini (2010) [17]	16 (37%)	3 (7%)	28	15	18 (42%)	32	34	32	34%	29%	42%
Hamaji (2013) [18]	47 (29.2%)	0	123	38	14 (8.7%)	72.9	NA	NA	60.8%	NA	NA
Ishikawa (2014) [19]	NA	0	54	39	6 (6.5%)	NA	NA	NA	87.0%	NA	92.5%
Xiao (2017) [20]	3 (5.8%)	0	38	14	21 (40.4%)	52	60	38	40.6%	NA	NA

Notes: *NA* Not available, *OS* Overall survival

### Results of quality assessment

NOS system was applied for quality assessment and all included articles scored no less than six stars (Table 1).

### Sublobar vs. standard group

**Fig. 2:**
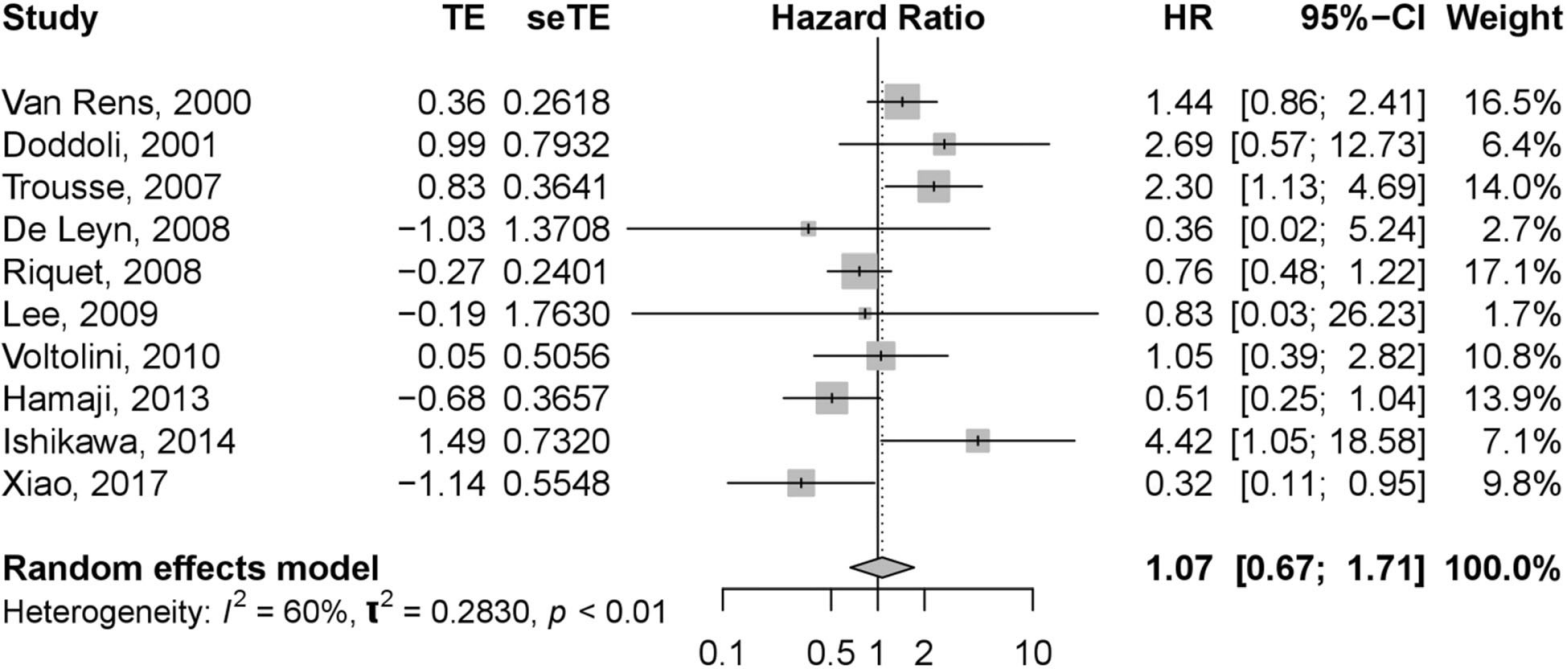
Forest plot of HR of OS in Sublobar vs. Standard resection group

The HR and 95% CI of OS of postoperative MPLC cases in sublobar group in comparison with standard group were extracted or calculated from each study. It was noted that the OS of metachronous MPLC cases started from the day of second operation. The heterogeneity of the pooled HR showed that *I*^2^ = 59.7%, *p* = 0.0079 < 0.10. Hence random-effect model was used for data analysis. The pooled HR of OS of MPLC cases in sublobar group relative to standard group was 1.07 (95%CI: 0.67–1.71), as shown in Fig. 2. The result suggested that the OS of MPLC patients undergoing anatomical lobectomy or pneumonectomy was of no statistical difference with those undergoing sublobar resection for at least one lesion.

### Subgroup analysis

**Fig. 3:**
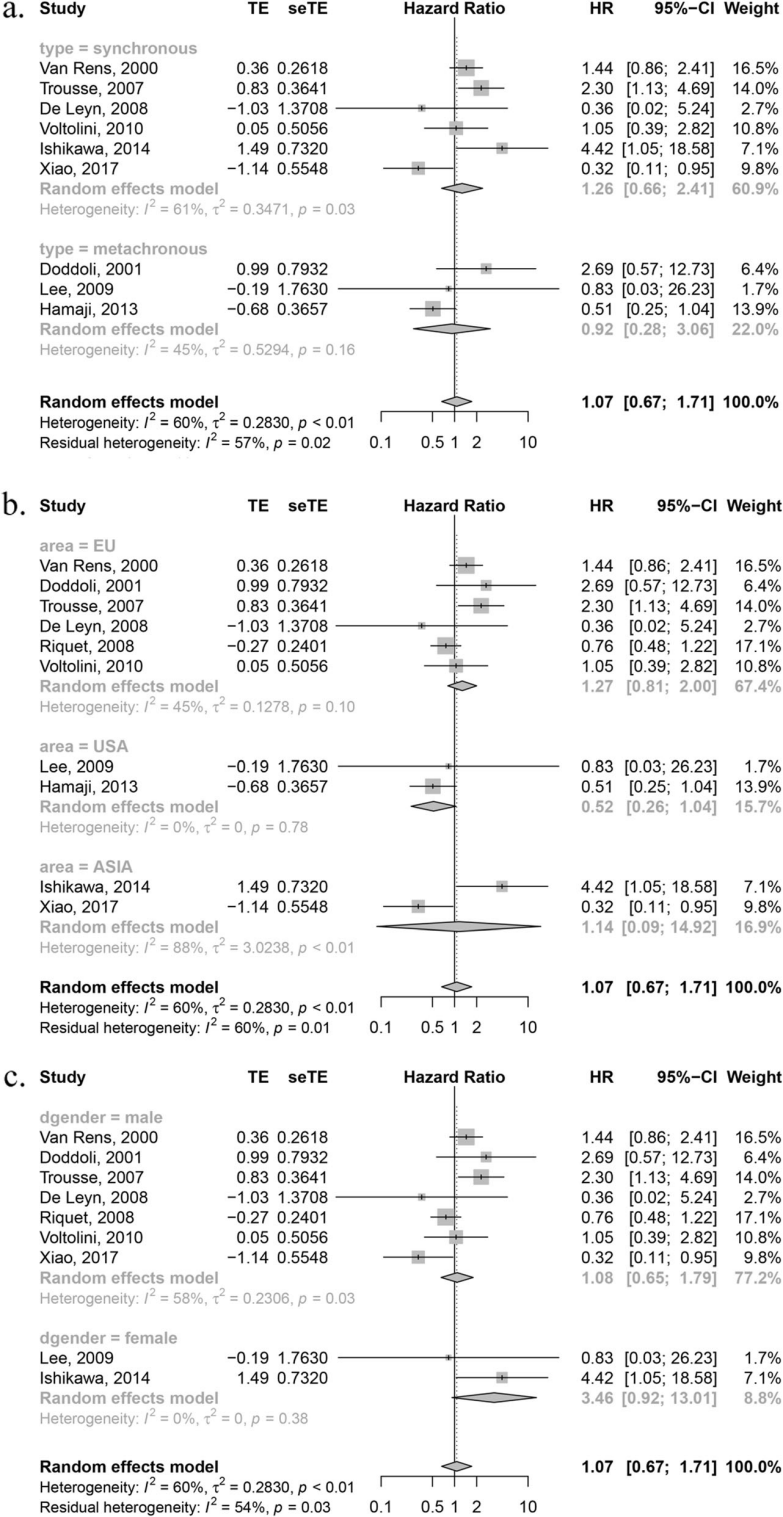
Subgroup analysis. **a** based on synchronous and metachronous type; **b** based on region; **c** based on dominant sex type

Subgroup analysis was carried out according to the type of MPLC (synchronous or metachronous), population region and dominant sex. As shown in Fig. 3, no statistically significant results were shown.

### Publication bias

**Fig. 4:**
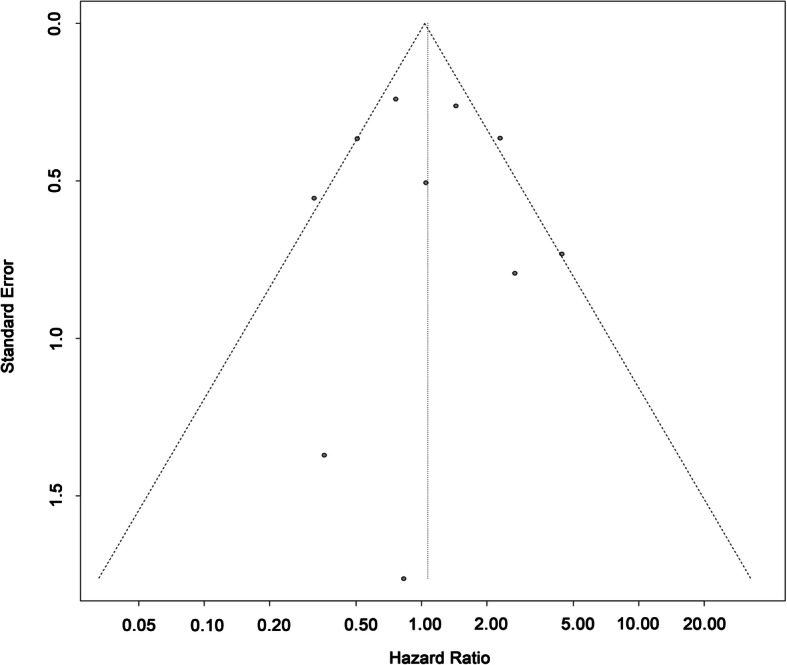
Publication bias (Funnel Plot)

As was displayed in Fig. 4, the funnel plot showed no significant asymmetry. And the results for Begg’s and Egger’s test were *p* = 0.788 > 0.05 and *p* = 0.874 > 0.05 respectively. These results indicated that there was no significant publication bias in this study.

## Discussion

Multiple primary lung cancer (MPLC) is a special type of lung cancer characterized by more than one cancerous lesions independent of each other and is becoming more common clinically. It may be widely accepted that surgery is the principal treatment for MPLC patients. For these multiple primary lesions, some applied radical resection for all while others used sublobar resection. As the surgical strategy for the treatment of MPLC is divergent, this study aimed to evaluate the difference between these two strategies in terms of prognosis. This study included 913 cases that went through resection of at least two lesions from ten independent researches. And the results suggested that sublobar resection was not an indicator for worse prognosis compared with complete standard resection for all lesions (HR: 1.07, 95%CI: 0.67–1.71). With medium heterogeneity, subgroup analysis based on different type (synchronous or metachronous), geographic origin and dominant sex was carried out, which showed no statistical difference. No publication bias was detected by either qualitative or quantitative methods.

As for single lesion, anatomic pulmonary resection is recommended for patients at early stages [7], which might be applicable to MPLC patients. Yet sublobar resection including segmentectomy and wedge resection is appropriate in selected patients. Indications include poor pulmonary reserve or other major comorbidities that contraindicate lobectomy, peripheral nodule less than 2 cm with at least one of the following: pure AIS, nodule has more than 50% ground-glass appearance on CT, or radiologic surveillance confirms a long doubling time (more than 400 days). Adenocarcinoma in situ (AIS) is defined as a small (≤3 cm) localized nodule with lepidic growth, mostly non-mucinous and multiple AIS tumors can occur synchronously [21]. Researches showed that for small size (less than 3 cm), low-risk and poor pulmonary function NSCLC patients, those taking sublobar resection had a comparable survival outcome compared with standard surgical approach [22]. A research by Fan et al. [23] found that for stage I NSCLC patients, those undergoing lobectomy tended to have better survival than sublobar resection; but the difference was insignificant while comparing stage Ia patients with lesion size less than 2 cm in diameter. Another research aiming at solid pulmonary nodules at stage Ia also indicated that these two surgical strategies had no significant difference in terms of patient survival [24]. Some researchers reckoned that for early stage NSCLC, sublobar resection, without improving peri-operative mortality, would increase the risk of non-R0 resection, decrease the number of lymph nodes dissected and thus influence the upstaging of N-stage, which would lead to worse prognosis and higher regional recurrence rate [25]. As for MPLC patients, anatomic lobectomy or even pneumonectomy may not be applied. For those with less pulmonary reserve, especially when multiple lesions locate in ipsilateral different lobes or contralateral lobes, standard operation is neither applicable nor safe. Most MPLC patients considering radical resection as main treatment strategy were considered to be at an early stage during pre-operative evaluation, especially when looking into the second large lesion independently. That is to say, it was possible that these lesions were of lower risk and suitable for sublobar resection. Hence no negative effects on patients’ prognosis were found applying the relatively conservative surgical strategy. This finding was in accordance with the impression during clinical practice in our center to treat MPLC patients surgically, as those taking sublobar resection strategy have not shown any tendency towards worse survival so far.

The result of the study showed medium heterogeneity, which limited the value of the combined HR. Though subgroup analysis had been applied, the sources of heterogeneity could not be disclosed completely. Besides, as all studies included were carried out retrospectively and randomization could not be obtained in observational studies, there could be selective bias. Therefore, the main limitation of this study was the relatively low quality of included literature. And the relatively strict inclusion and exclusion criteria led to a small number of included studies. Also, the publication language was limited to English for the improvement of literature quality, which inevitably increased publication bias.

Although TNM staging play a crucial role in the prediction of the prognosis of NSCLC patients, we were not able to apply it for analysis in this research due to different versions of staging system adopted in each literature. A few researchers found that the highest pT stage of MPLC was an independent prognostic factor [16, 20]. Some researches indicated that lymph node involvement was an indicator for prognosis [13, 19] while others found no significant correlation between the two [17, 18]. Overall, there tended to be a correlation between TNM stage situation of MPLC and postoperative survival. However, because of the lack of unanimity in the classification of multiple primary lung cancer [26], we could not draw any conclusion and further researches on this particular subject were needed.

A few researches suggested that stage I NSCLC patients with single lesion receiving segmentectomy tended to have better prognosis compared with wedge resection [27, 28]. As these ten researches did not compare the difference between these two approaches, no further analysis could be made here. A more specific and precise comparison of various sublobar resection approaches need to be further studied.

For synchronous MPLC lesions adopting sequential resection, De Leyn et al. [14] preferred to have the larger lesion removed first, while Trousse et al. [13] held that the smaller one should be dealt with first for safety and a better chance for second operation. Researchers believed that synchronous MPLC with ipsilateral sites should apply lung function preserving approaches, i.e. sublobar resection, while avoiding pneumonectomy; and those with contralateral sites should adopt staged operation [4]. Yet no evidence was given in terms of the sequence of resections.

## Conclusion

In conclusion, this study may reveal that sublobar resection is acceptable for patients with MPLC at an early stage, because of the equivalent prognosis as the standard resection and better pulmonary function preservation. However, further research is needed to validate these findings.

## Data Availability

The datasets used and analyzed during the current study are available from the corresponding author on reasonable request.

## Abbreviations

ACCP: American College of Chest Physicians
ADK: Adenocarcinoma
AIS: Adenocarcinoma In Situ
BTS: British Thoracic Society
CI: Confidence Interval
CSCO: Chinese Society of Clinical Oncology
GGO: Ground Glass Opacity
HR: Hazard Ratio
MET: Metachronous
MPLC: Multiple Primary Lung Cancer
NCCN: National Comprehensive Cancer Network
NOS: the Newcastle-Ottawa Scale
NSCLC: Non-Small Cell Lung Cancer
OS: Overall Survival
PRISMA: Preferred Reporting Items for Systematic Reviews and Meta-Analyses
SCC: Squamous Cell Carcinoma
SE: Standard Error
SYN: Synchronous

## References

[1] Murphy SJ, Aubry MC, Harris FR, Halling GC, Johnson SH, Terra S, Drucker TM, Asiedu MK, Kipp BR, Yi ES (2014). Identification of independent primary tumors and intrapulmonary metastases using DNA rearrangements in non-small-cell lung cancer. J Clin Oncol.

[2] Martini N, Melamed MR (1975). Multiple primary lung cancers. J Thorac Cardiovasc Surg.

[3] Kozower BD, Larner JM, Detterbeck FC, Jones DR (2013). Special treatment issues in non-small cell lung cancer: diagnosis and management of lung cancer, 3rd ed: American College of Chest Physicians evidence-based clinical practice guidelines. Chest.

[4] Waller DA (2018). Surgical management of lung cancer with multiple lesions: implication of the new recommendations of the 8(th) edition of the TNM classification for lung cancer. Journal of thoracic disease.

[5] Hamaji M, Ali SO, Burt BM (2015). A meta-analysis of resected metachronous second non-small cell lung cancer. Ann Thorac Surg.

[6] Leventakos K, Peikert T, Midthun DE, Molina JR, Blackmon S, Nichols FC, Garces YI, Hallemeier CL, Murphy SJ, Vasmatzis G (2017). Management of Multifocal Lung Cancer: results of a survey. J Thorac Oncol.

[7] NCCN Clinical Practice Guidelines in Oncology: Non-Small Cell Lung Cancer. 2019. https://www.nccn.org/professionals/physician_gls/pdf/nscl.pdf. Accessed 18 Jan 2019.

[8] Jiang G, Chen C, Zhu Y, Xie D, Dai J, Jin K, Shen Y, Wang H, Li H, Zhang L et al: [Shanghai pulmonary hospital experts consensus on the Management of Ground-Glass Nodules Suspected as lung adenocarcinoma (version 1)]. Zhongguo Fei Ai Za Zhi 2018, 21(3):147–159. 10.3779/j.issn.1009-3419.2018.03.05 PMC5973030 29587930

[9] Liberati A, Altman DG, Tetzlaff J, Mulrow C, Gotzsche PC, Ioannidis JP, Clarke M, Devereaux PJ, Kleijnen J, Moher D (2009). The PRISMA statement for reporting systematic reviews and meta-analyses of studies that evaluate healthcare interventions: explanation and elaboration. Bmj.

[10] Parmar MK, Torri V, Stewart L (1998). Extracting summary statistics to perform meta-analyses of the published literature for survival endpoints. Stat Med.

[11] van Rens MT, Zanen P, Brutel de La Riviere A, Elbers HR, van Swieten HA, van Den Bosch JM (2000). survival in synchronous vs. single lung cancer: upstaging better reflects prognosis. Chest.

[12] Doddoli C, Thomas P, Ghez O, Giudicelli R, Fuentes P (2001). Surgical management of metachronous bronchial carcinoma. European journal of cardio-thoracic surgery : official journal of the European Association for Cardio-thoracic Surgery.

[13] Trousse D, Barlesi F, Loundou A, Tasei AM, Doddoli C, Giudicelli R, Astoul P, Fuentes P, Thomas P (2007). Synchronous multiple primary lung cancer: an increasing clinical occurrence requiring multidisciplinary management. J Thorac Cardiovasc Surg.

[14] De Leyn P, Moons J, Vansteenkiste J, Verbeken E, Van Raemdonck D, Nafteux P, Decaluwe H, Lerut T (2008). Survival after resection of synchronous bilateral lung cancer. Eur J Cardio-Thoracic Surg : Official J Eur Association Cardio-thoracic Surg.

[15] Riquet M, Cazes A, Pfeuty K, Ngabou UD, Foucault C, Dujon A, Banu E (2008). Multiple lung cancers prognosis: what about histology?. Ann Thorac Surg.

[16] Lee BE, Port JL, Stiles BM, Saunders J, Paul S, Lee PC, Altorki N (2009). TNM stage is the most important determinant of survival in metachronous lung cancer. Ann Thorac Surg.

[17] Voltolini L, Rapicetta C, Luzzi L, Ghiribelli C, Paladini P, Granato F, Gallazzi M, Gotti G (2010). Surgical treatment of synchronous multiple lung cancer located in a different lobe or lung: high survival in node-negative subgroup. Eur J Cardio-thoracic Surg : Official J Eur Association Cardio-thoracic Surg.

[18] Hamaji M, Allen MS, Cassivi SD, Deschamps C, Nichols FC, Wigle DA, Shen KR (2013). Surgical treatment of metachronous second primary lung cancer after complete resection of non-small cell lung cancer. J Thorac Cardiovasc Surg.

[19] Ishikawa Y, Nakayama H, Ito H, Yokose T, Tsuboi M, Nishii T, Masuda M (2014). Surgical treatment for synchronous primary lung adenocarcinomas. Ann Thorac Surg.

[20] Xiao F, Liu D, Liang C (2017). Survival and prognostic factors of synchronous multiple primary NSCLC and further differentiation from intrapulmonary metastasis. J Thorac Oncol.

[21] Travis WD, Brambilla E, Noguchi M, Nicholson AG, Geisinger KR, Yatabe Y, Beer DG, Powell CA, Riely GJ, Van Schil PE (2011). International association for the study of lung cancer/american thoracic society/european respiratory society international multidisciplinary classification of lung adenocarcinoma. J Thorac Oncol.

[22] Nakamura H, Kawasaki N, Taguchi M, Kabasawa K (2005). Survival following lobectomy vs limited resection for stage I lung cancer: a meta-analysis. Br J Cancer.

[23] Fan J, Wang L, Jiang GN, Gao W (2012). Sublobectomy versus lobectomy for stage I non-small-cell lung cancer, a meta-analysis of published studies. Ann Surg Oncol.

[24] Altorki NK, Yip R, Hanaoka T, Bauer T, Aye R, Kohman L, Sheppard B, Thurer R, Andaz S, Smith M (2014). Sublobar resection is equivalent to lobectomy for clinical stage 1A lung cancer in solid nodules. J Thorac Cardiovasc Surg.

[25] Khullar OV, Liu Y, Gillespie T, Higgins KA, Ramalingam S, Lipscomb J, Fernandez FG (2015). Survival after sublobar resection versus lobectomy for clinical stage IA lung Cancer: an analysis from the National Cancer Data Base. J Thorac Oncol.

[26] Fonseca A, Detterbeck FC (2014). How many names for a rose: inconsistent classification of multiple foci of lung cancer due to ambiguous rules. Lung cancer (Amsterdam, Netherlands).

[27] Koike T, Koike T, Yoshiya K, Tsuchida M, Toyabe S (2013). Risk factor analysis of locoregional recurrence after sublobar resection in patients with clinical stage IA non-small cell lung cancer. J Thorac Cardiovasc Surg.

[28] Sihoe ADL, Van Schil P (2014). Non-small cell lung cancer: When to offer sublobar resection. Lung cancer (Amsterdam, Netherlands).

